# Current practices underestimate environmental exposures to methamphetamine: inhalation exposures are important

**DOI:** 10.1038/s41370-020-00260-x

**Published:** 2020-09-01

**Authors:** Jackie Wright, Bob Symons, Jonathon Angell, Kirstin E. Ross, Stewart Walker

**Affiliations:** 1 Health and Environment, College of Science and Engineering, Flinders University, GPO Box 2100, Adelaide, SA 5001 Australia; 2 Environmental Risk Sciences Pty Ltd, PO Box 2537, Carlingford Court, NSW 2118 Australia; 3 Eurofins, 1/21 Smallwood Place, Murarrie, QLD 4172 Australia; 4 Physical and Chemical Sciences, College of Science and Engineering, Flinders University, GPO Box 2100, Adelaide, SA 5001 Australia

**Keywords:** Methamphetamine, Underestimate, Environmental exposure, Inhalation, Contaminant transfer

## Abstract

Current practice for determining the exposure to methamphetamine in contaminated homes relies on the analysis of surface wipe sample to address direct contact exposures. The movement of methamphetamine into the air phase, and the potential for inhalation exposures to occur within residential homes contaminated from former clandestine manufacture or smoking of methamphetamine has been generally poorly characterised and understood. All available risk-based guidelines for determining safe levels of methamphetamine in residential properties do not include any consideration of the inhalation pathway as an exposure route. This study showed that methamphetamine can readily move from contaminated materials in a home into the air phase. This movement of methamphetamine into the air phase provides both an exposure pathway and a mechanism for the transfer of methamphetamine throughout a property. The inhalation exposure pathway has the potential to result in significant intake of methamphetamine, adding to dermal absorption and ingestion exposure routes. Guidelines that are established for the assessment of methamphetamine contaminated properties that ignore inhalation exposures can significantly underestimate exposure and result in guidelines that are not adequately protective of health. This study also demonstrates that sampling methamphetamine in air can be undertaken using commercially available sorption tubes and analytical methods.

## Introduction

The clandestine manufacture of methamphetamine, as well as the smoking of crystal methamphetamine (commonly referred to as “ice”), results in the contamination of properties [1, 2]. Of particular concern is the presence of methamphetamine residues within the property, which can remain for a long period of time if the property is not remediated. People working or living in these contaminated properties are exposed to these methamphetamine residues, and significant adverse health effects have been reported [1, 3]. A number of countries and jurisdictions have developed health-risk-based guidelines for the assessment and remediation of methamphetamine residues in properties [4–8]. These guidelines are based on the protection of public health, with one guideline value adopted for determining whether a property is considered to be contaminated as well as being the target level for ‘successful’ remediation. These guidelines are based on the underlying assumption that methamphetamine residues remain as residues on surfaces, where exposure occurs via dermal contact and ingestion of residues from hands or objects. None of the guidelines currently available consider, or include, the inhalation pathway as it is assumed that methamphetamine is not sufficiently volatile for this pathway to be of significance, particularly post remediation [5].

Although methamphetamine hydrochloride is non-volatile, methamphetamine may be present in the air that is breathed as the more volatile free base or absorbed to or present on particles (dust). These sources are referred to in this paper as ‘air phase’. Methamphetamine has been detected in air during controlled manufacture [9–14] as this is the key mechanism for residues to be released during manufacture and spread throughout a premises. During different phases of manufacture, methamphetamine has been detected in air at levels up to 42 µg/m^3^ during the cook phase, 5500 µg/m^3^ during salting out, 5500 µg/m^3^ immediately post cook and 210 µg/m^3^ 24-h post manufacture for indoor cooking [9–14]. When methamphetamine is smoked, levels between 300 and 1600 µg/m^3^ have been reported [15]. Sampling conducted during these controlled manufacture and smoke events involved pumping air through acid-treated glass fibre filters and analysis of the filters using an appropriate method by gas chromatography mass spectrometry (GC or GC-MS) or liquid chromatography mass spectrometry (HPLC or LC-MS). This methodology characterised the presence of methamphetamine aerosols present in air, not necessarily methamphetamine in the vapour (gaseous) phase.

In contrast air sampling conducted by the Minnesota Pollution Control Agency [16, 17] aimed to specifically distinguish between aerosol (as either methamphetamine base and methamphetamine hydrochloride) and vapour phase methamphetamine (assumed to be methamphetamine base) in indoor air environments. This involved the use of a pump to draw air through a glass fibre filter to target the semi-volatile or particulate phase and then through an acid-treated silica gel sorbent tube to target the volatile phase, with analysis by LC-MS.

Methamphetamine has been reported in indoor air at levels between 0.2 and 7.3 µg/m^3^ in properties seized by police in the US for clandestine manufacture [9, 12]. This sampling involved pumping air through acid-treated glass fibre filters and analysis of the filters using GC-MS.

Solid phase microextraction (SPME) has been used to trap methamphetamines from the air for subsequent analysis by GC-MS. Volatilisation, trapping and analysis of methamphetamine hydrochloride as methamphetamine free base with on-fibre derivatisation by a range of chloroformates on a range of SPME fibres have been shown to be effective in recovering methamphetamine (and precursors and by-products) from the headspace of gloves and money recovered from a clandestine laboratory [18]. Levels between 0.2 and 3 µg/m^3^ have been reported in properties suspected to have been formerly used to manufacture methamphetamine in New Zealand, only where surface methamphetamine contamination exceeded 40 µg/100 cm^2^ [19]. This method involved the sampling of methamphetamine vapours using a laboratory based method with a SPME field sampler and analysis by GC-MS [20]. This methodology has been further refined [21, 22] adapting a method using a capillary microextraction (CME) device with larger surface area [23] to enable a lower level of detection to be achieved. However, both sampling methods require analysis within 5 days and neither method is commercially available. The CME method has not been used to sample indoor air in methamphetamine contaminated properties. While the CME methodology is cost effective it is not currently considered to be a practical method for sampling in a range of indoor environments to evaluate the significance, or otherwise, of the methamphetamine inhalation pathway.

While these methods were reported to be reliable during test sampling, little data is available from indoor air environments from former clandestine drug laboratories and the methods are not commercially available. Therefore, this study has been undertaken to further evaluate commercially available methods for the sampling and analysis of methamphetamine in air, and to evaluate the potential for the inhalation pathway to be of significance in properties known to be contaminated from manufacture and/or use.

## Materials and methods

### General

The sampling of ambient air was undertaken using commercially available ORBO^TM^-49P (OVS) Supelpak™-20 sampling tubes. These tubes are packed with treated Amberlite^®^ XAD^®^-2 resin and were designed for sampling organophosphorus pesticides, specifically chlorpyrifos, diazinon, dichlorvos, malathion and parathion in both vapour (gaseous) and aerosol form, with the packing material used effectively absorbing and releasing these compounds for analysis. These pesticides, along with methamphetamine and methamphetamine hydrochloride are semi-volatile organic chemicals, with vapour pressures in the same range. These attributes led to the selection of this media for this study.

Sampling of air using these tubes involved the use of a personal sampling pump (SKC Airchek Sampler PCXR4) and clean flexible tubing to connect the sample tube to the pump. The sampling rate was set at ~1 L/min with the total volume sampled kept to a total of <480 L, consistent with NIOSH 5601 (NIOSH 2016) and OSHA Method 62 (OSHA 1986) for the sampling of organonitrogen pesticides. To evaluate the suitability of the tubes and the analytical method for the reliable reporting of methamphetamine in air, the sampling programme included a field blank (FB), internal standard recoveries, laboratory spike (LS) samples and field spike samples.

The tubes were analysed by Eurofins in Brisbane, Australia, using a commercially available method based upon NIOSH Methamphetamines—Method 9111 [24] that is used for the analysis of wipe samples. The extraction for methamphetamine, amphetamine, pseudoephedrine, ephedrine, methylenedioxymethamphetamine (MDMA) and 3,4-methylenedioxyamphetamine (MDA) used 0.1-M sulfuric acid made up in methanol, and the desorption was aided by vortexing the mixture of XAD-2 resin between 3 and 5 min and then making up to a 30-mL volume in methanol. The extraction efficiency was determined by adding 1.5 µg of deuterated-labelled analogues of the native drugs viz d,l-amphetamine-D5.hyrochloride, d,l-methamphetamine-D5.hydrochloride, d,l-MDMA-D5.hydrochloride, d,l-MDA-D5.hydrochloride and d-ephedrine-D3.hydrochloride (+)-ephedrine-D3.hydrochloride prior to extraction as well as spiking of blank ORBO^TM^-49P (OVS) Supelpak™-20 sampling tubes with the native drugs. Furthermore, field spikes were conducted whereby the unused sampling tubes were spiked in the laboratory with 1.5 µg of deuterated-labelled analogues prior to sampling and then transported into the field and then sampling undertaken. This methodology has a stated maximum holding time of 30 days between sample collection and analysis.

Analysis of native and deuterated-labelled analogues of the target drugs was conducted using liquid chromatography coupled with tandem mass spectrometry (LC-MS/MS). The LC-MS/MS system comprised an Agilent 1290 Infinity UHPLC system with autosampler, degasser and refrigeration unit and an Agilent 6460/6470 Triple Quadrupole LC/MS with Jet Stream Technology and electrospray source. Separation of the compounds was accomplished using either a Phenomenex Kinetex Biphenyl Column 3.0 × 50 mm, 2.6-μm particle size, or Agilent ZORBAX Eclipse Plus C18 Column 2.1 × 50 mm, 1.8-μm particle size columns. It should be noted that the NIOSH Method 9111 is operated in selected ion monitoring mode using only a single quadrupole mass spectrometer whereas the method described in this paper uses positive-ion multiple reaction monitoring whereby several specific transitions were monitored for each compound for maximum selectivity and sensitivity. The analytical method validation and verification parameters for the reporting of amphetamine and methamphetamine, pseudoephedrine, ephedrine, MDMA and MDA are included in Table 1.

**Table 1:** Method validation/verification parameters.

Analytical parameter	MDL^a^ (µg)	LLOR^b^ (µg)	Target LOR (µg)	MU^c^ (*k* = 2)	Precision	Accuracy
Ephedrine	0.0004	0.0012	0.02	20.3%	8.2%	86%
Pseudoephedrine	0.0001	0.0004	0.02	23.9%	5.2%	92%
Amphetamine	0.0003	0.0008	0.02	20.2%	5.2%	93%
Methamphetamine	0.0002	0.0005	0.02	20.2%	1.7%	93%
MDA	0.0002	0.0007	0.02	20.5%	4.4%	93%
MDMA	0.0002	0.0005	0.02	20.2%	4.3%	109%

^a^USEPA Method Detection Limit Procedure, Revision 2, December 2016 [25].

^b^LLOR—lower limit of reporting defined as 3.14 times the MDL.

^c^MU—expanded measurement uncertainty with a coverage factor (*k*) of 2.

### Field sampling

Although there are many homes contaminated with methamphetamine due to synthesis or smoking, the opportunity to undertake prolonged studies is rare. This paper reports on two locations that due to circumstances (e.g., prolonged legal dispute during which time the property was sealed and vacant—and known time of cessation of synthesis) longitudinal time studies could be performed. Air samples were collected by the first author from two locations in Australia where environmental methamphetamine contamination was known to be present. All samples were collected using a calibrated SKC pump, set to 1-L/min flow rate with clean flexible tubing used to connect the sample tube to the pump. It is noted that the air sampled passed through the sampling tube first, then the flexible tubing and then the pump.

The volume of air collected in each sample varied between 88 and 286 L. The variability in the sample volume collected reflected the limitations in access time available at each property.

House 1 (H1): This was an urban residential home where methamphetamine was suspected to have been manufactured 1 year prior to the current owners moving in and where they have resided for a period of 9 years prior to discovery of the presence of contamination. The family, particularly the two young children, was unwell while living in the home. Methamphetamine residues were reported on surfaces in the home at levels between 0.52 and 49 µg/100 cm^2^ on painted gyprock walls and an average on all surfaces that residents may regularly come into contact with 12.6 µg/100 cm^2^, with 250 µg/100 cm^2^ reported on the front of the split system air conditioning unit. The property had not been remediated but was vacant at the time of sampling. Air sampling was initially conducted with the air conditioning running as this was initially through to be a worst-case exposure scenario. The air conditioning unit was located in the dining room in an open plan area adjacent to the kitchen and lounge areas. Further sampling was also undertaken in other locations in the home, after the use of a portable air filtration unit (used in an attempt to try to clean the air in the house), with and without the air conditioner running. The samples collected from this home were to determine the potential significance of the inhalation pathway for methamphetamine contaminated properties that have not been remediated. The samples collected are summarised in Table 2.

**Table 2:** Air samples collected from contaminated properties.

Sample	Sample date	Description	Sample volume
House 1			
H1-IAD	February 2019	Sample from dining table located under air conditioning unit—air conditioning unit turned on	286 L
H1-IADX	March 2019	Sample from dining table located under air conditioning unit—air conditioning unit turned off	136.4 L
H1-IAKX	March 2019	Sample from children’s room (room furthest from air conditioner)—air conditioning unit turned off	148.8 L
H1-IAK	March 2019	Sample from children’s room—air conditioning unit turned on	144 L
H1-IAP	March 2019	Sample from kitchen/playroom doorway (adjacent to dining room)—air condition unit turned on	132 L
House 2			
H2-C	February 2019	Sample from a ventilated shipping container with contaminated possessions removed from the shed, noted to predominantly hard items or items stored in plastic boxes	88 L
H2-T1	February 2019	Samples (T1 and T2) from sealed bag containing soft toys removed from the residential house	144 L
H2-T2	April 2019 (tubes prepared as field spikes)		115 L
H2-SM	April 2019 (tubes prepared as field spikes)	Sample collected from sealed bag containing soft foam material removed from the residential house	115 L
QA samples			
FB	March 2019	Field blank	
ISR	February, March and April 2019	Internal standard recoveries (surrogate recoveries on all samples)	
LCS	February and March 2019	Laboratory control samples (analysed by the laboratory for each batch analysed in February and March 2019)	
LB	March 2019	Laboratory method blank (one sample)	
LS	March 2019	Laboratory spike sample (one sample)	
Field spikes	April 2019	Laboratory prepared the sample tubes with spikes prior to sampling, with recoveries reported at the completion of analysis (two samples)	

House 2 (H2): This was a rural property where methamphetamine manufacture was known to have occurred at some point in time prior to the home being sold to a family. The family lived in the property for 2 years prior to discovering the property was contaminated and was unwell while living in the home. Methamphetamine residues were reported on surfaces in the home at levels between 0.54 and 110 µg/100 cm^2^ on painted gyprock walls, with an average of 31 µg/100 cm^2^. At the time of sampling, the property was in the process of remediation; however, access had been provided to collect and store contaminated property and possessions prior to remediation commencing. Air samples were collected from a shipping container that stored property removed from the shed, from a sealed bag of soft toys taken from the home, and from a sealed bag of foam materials removed from the home. The sampling undertaken for this property was to determine if methamphetamine had the potential to move into the air phase from possessions previously stored in the methamphetamine contaminated property. The samples collected are summarised in Table 2.

## Results

### Data quality

Method detection limits (MDL), summarised in Table 1, were calculated in accordance with USEPA Method Detection Limit Procedure, Revision 2, December 2016 [25] and ranged between 0.0001 and 0.0004 μg for pseudoephedrine and ephedrine, respectively. For the key analytes considered in this paper the MDLs were 0.0002 μg for methamphetamine and 0.0003 μg for amphetamine. From the MDL, lower limits of reporting were calculated as 3.14 times the MDL. Target limit of reporting was set at 0.02 μg for each of the six analytes. The linear calibration curve is 0.002, 0.024, 0.150, 0.302, 1.465 and 3.018 μg, with 3.018 μg being the upper limit for each of the six analytes. Any compounds that were above the upper limit were diluted to be approximately mid-point on the linear calibration curve to be quantified.

Measurement uncertainty with coverage factor (*k*) of 2 ranged from 20.2 to 23.9%. Precision ranged from 1.7% for methamphetamine and 8.2% for ephedrine and accuracy ranged between 86% for ephedrine and 109% for MDMA.

There were no detections reported on the FB or the laboratory method blanks. The LS and laboratory control samples (Table 3) for the six analytes came in with a range of 99–112% and 93–124% with methamphetamine being 112 and 103–107%, respectively, and amphetamine being 106 and 102–109%, respectively. The field spike samples also came within 84–103% for methamphetamine and 74–108% for amphetamine. The surrogate recoveries on the individual samples analysed were generally within the range of 70–130% for methamphetamine and amphetamine, with the exception of a few samples where the surrogate recoveries were just outside this range.

**Table 3:** Spike/surrogate recoveries.

Analyte reported	Recoveries (%)			
	Lab spike (LS) (March 2019)	Laboratory control samples (LCS) (February and March 2019)	Surrogate recoveries (ISR) (February, March and April 2019)	Field spike samples (April 2019)
Methamphetamine	112%	103–107%	69–148%	84–103%
Amphetamine	106%	102–109%	59–126%	74–108%
Pseudoephedrine	99%	93–116%	74–120%	Not reported
Ephedrine	102%	97–124%	74–120%	81–103%
MDMA	104%	101–107%	69–148%	78–98%
MDA	106%	100–106%	58–133%	83–88%

Based on the data relating to analytical validation and laboratory and field parameters, the data quality is considered to be acceptable, showing that the sampling tubes and analytical method can achieve acceptable levels of recoveries for the drugs evaluated, specifically methamphetamine and amphetamine.

### Analytical results

Pseudoephedrine, ephedrine, (both indicators of synthesis) and MDMA and MDA (indicators of poly drug use) were not detected in any of the air samples collected and have not been further discussed. Methamphetamine was detected in all samples collected, with amphetamine detected in some samples. Table 4 presents the methamphetamine and amphetamine air concentrations reported in each of the samples. Owing to the large range of concentrations encountered in the different houses (3 orders of magnitude) the values for methamphetamine and amphetamine in Table 4 are quoted to two significant figures.

**Table 4:** Air results.

Sample location and ID	Methamphetamine (µg/m^3^)	Amphetamine (µg/m^3^)
House 1		
H1-IAD	0.53	0.013
H1-IADX	4.5	0.29
H1-IAKX	3.8	0.20
H1-IAK	6.2	0.21
H1-IAP	8.3	0.30
House 2		
H2-C	0.008	Not detected
H2-T1	0.046	Not detected
H2-T2	0.30	0.013
H2-SM	0.0016	0.0087

## Discussion

The sampling of air from inside a home, or from air around materials taken from a home known to be contaminated with methamphetamine residues, indicates that there is a measurable level of methamphetamine and amphetamine present in the air phase. Where these drugs are present in the air-phase indoors, residents living in these homes will be exposed via inhalation.

In addition, the presence of methamphetamine in air provides a mechanism for contamination to move and be transferred throughout a property well after the end of manufacture or use. The movement of methamphetamine from gyprock/gypsum wall materials to air has also been reported in laboratory studies [26, 27], where an equilibrium partition coefficient was established. The movement of methamphetamine from gyprock/gypsum wall materials into the air phase was found to increase with increased temperature and increased relative humidity (particularly at higher temperatures) [26]. It is noted that these conditions (high temperature and high humidity) not only cause increased concentration of methamphetamine into the air phase but will also result in the increased skin exposure as people (especially children) will wear less clothes under these climatic conditions. The absorption of methamphetamine into wall materials, and other materials in a property, and the potential further desorption and transfer or movement of methamphetamine throughout a property has also been reported for H2 [28].

The sampling of air using commercially available sorbent tubes and analytical methods, as detailed in this paper, provides reliable results with acceptable levels of recovery.

### House 1 (H1)

Despite the significant amount of time between suspected manufacture and the collection of air samples in H1, concentrations of methamphetamine in air were up to 8.3 µg/m^3^. This concentration is one to two orders of magnitude below levels reported in air during the cook or only 24-hour post cook [9–14], and consistent with levels in air reported in properties seized by police in the US for active manufacture [9, 12].

To better understand the potential significance of the inhalation pathway, compared with dermal absorption and ingestion, potential intakes of methamphetamine have been calculated for adults and young children living in H1.

Based on the approach adopted in the development of methamphetamine surface residue guidelines in Australia [4, 29], intakes of methamphetamine via dermal absorption through the skin (for hands and the rest of the body) and ingestion of methamphetamine residues from hands and objects (for children only) have been calculated. The assumptions adopted for the amount of skin that may be exposed for children and adults are sufficiently representative of activities that may occur in most of Australia, however in hotter climatic areas the exposed skin surface area may be an underestimate. In addition potential intakes of methamphetamine via inhalation has been undertaken on the basis of Australian and international guidance [30, 31], assuming exposures occur inside the home for 20 hours each day, for 365 days per year [30] with young children inhaling 4.95 m^3^ air/day (average for children aged 1–2 years [32]) and adults inhaling 15 m^3^ air/day [30]. Where these calculations are undertaken on the basis of the average level of methamphetamine residues on surfaces in the home (12.6 µg/100 cm^2^) and the average concentration in air (4.7 µg/m^3^), Table 5 presents the intakes calculated for each pathway, along with the total intake from all pathways inside the home. The table also includes the reference dose (RfD) for methamphetamine adopted on the development of surface residue guidelines in Australia and California [33].

**Table 5:** Calculated intakes of methamphetamine relevant to living in House 1.

Exposure pathway	Intake of methamphetamine (mg/kg/day)	
	Young children	Adults
Dermal absorption from hands	0.002	0.0004
Dermal absorption from rest of the body	0.005	0.0001
Ingestion from residues on hands	0.0003	0.00003
Ingestion from residue on objects	0.00009	Not applicable for adults
Inhalation	0.002	0.0008
Total intake (all pathways)	0.01	0.001
Reference dose	0.0003	0.0003

Table 5 indicates that for both adults and children, intakes of methamphetamine via the inhalation pathway alone exceed the RfD. Where all exposures are considered, the total intake also exceeds the RfD.

For young children, intakes of methamphetamine are dominated by the dermal absorption pathways as young children come into regular contact with surfaces and objects in a home. Inhalation exposures account to 20% of the total intake. This is a significant proportion of all intakes of methamphetamine in the home.

For adults, intakes of methamphetamine from dermal absorption are lower as adults do not come into regular extensive contact with surfaces and objects in the home. In this situation intakes via inhalation comprise 59% of the total intake. This is a more significant pathway for adults.

For both adults and children, there is likely to be some variability in intakes that may be derived from dermal absorption and ingestion of methamphetamine. However, inhalation exposures will always occur whenever living in the property. From the data collected from H1, this suggests that the inhalation pathway is a significant exposure pathway, particularly for adults where intakes from other exposure pathways is lower than for young children. While this data is from one property, the data indicates that not accounting for the inhalation of methamphetamine in properties that have not been remediated may significantly underestimate potential exposures.

### House 2 (H2)

The air samples collected from H2 differ from those collected in H1. It was not possible to collect air samples from indoor air spaced in the residential home as the property was being remediated at the time. The samples collected show that methamphetamine moves into the air phase from property and possessions removed from the contaminated home.

The property including possessions have been sampled and previously reported [28]. Methamphetamine was reported on personal possessions taken into the contaminated home at least a year after the end of methamphetamine synthesis, indicating that there was the transfer of methamphetamine throughout the home. A study by Morrison et al. [34] demonstrated the sorption of methamphetamine from the air phase onto porous textiles that include clothing and soft toys. Hence once in the air phase, methamphetamine can further sorb into other materials.

Air concentrations reported from the bag containing the soft toys ranged from 0.046 to 0.3 µg/m^3^, with an approximate tenfold difference in concentration over the two sampling events. The reason for the difference in concentration is not known; however, it may be due to better sealing of the bag from which the sample was collected during the second sampling event or different temperatures at the time of sampling, noting that temperature was not recorded.

Methamphetamine levels from former analysis of soft toys, consistent with the items in the bag from which the air samples were collected, were in the range of 1.09–12.2 mg/kg.

For the bag containing the foam material, a lower air concentration of 0.0016 µg/m^3^ was reported, consistent with a lower concentration of methamphetamine reported in previous analysis of similar foam material (1.6 mg/kg). These data show that when stored in a sealed environment methamphetamine present in the soft materials will continue to move into the air phase.

This data further supports that the air phase is an important aspect of the transfer of methamphetamine contamination and home and the inhalation pathway is relevant when evaluating exposures that may occur.

### Study limitations

This study has focused on the use of commercially available sorbent tubes and analytical methods to provide an indication of air concentrations of methamphetamine in actual properties. The methodology has not included an assessment of the recovery of methamphetamine in the gas phase using the sorbent tubes selected. This, along with comparative studies using other methods should be evaluated in future studies.

## Conclusions

This study clearly shows that methamphetamine can readily move from contaminated materials in a home into the air phase where inhalation will occur. This movement of methamphetamine into the air phase is also a key mechanism for the transfer or movement of contamination throughout a property. The inhalation exposure pathway has the potential to result in significant intakes of methamphetamine, adding to intakes that are more well known to occur that include dermal absorption and ingestion. Where guidelines are established for the assessment of methamphetamine contaminated properties, or properties contaminated with other illicit drugs, ignoring inhalation exposures (as is currently undertaken) can significantly underestimate exposure and result in guidelines that are not adequately protective of health.

This study also supports that the sampling of methamphetamine in air can be undertaken using commercially available sorption tubes and analytical methods. This will allow for easier and potentially more regular sampling of indoor air in a range of properties so that the findings of this study can be further evaluated in a range of different situations.

This study shows that current practices underestimate the environmental exposure risk for people living in contaminated homes when only a surface wipe sample (10 × 10 cm in area) is considered. The amount of exposure due to inhalation on air-phase methamphetamine needs to be included to determine the real exposure risk.
